# DAC can restore expression of NALP1 to suppress tumor growth in colon cancer

**DOI:** 10.1038/cddis.2014.532

**Published:** 2015-01-22

**Authors:** C Chen, B Wang, J Sun, H Na, Z Chen, Z Zhu, L Yan, S Ren, Y Zuo

**Affiliations:** 1 Department of Clinical Biochemistry, Dalian Medical University, Dalian 116044, China; 2 Department of General Surgery, The Second Affiliated Hospital of Dalian Medical University, Dalian 116023, China

## Abstract

Despite recent progress in the identification of genetic and molecular alternations in colorectal carcinoma, the precise molecular pathogenesis remains unclear. NALP1 (nucleotide-binding oligomerization domain-like receptor family, pyrin domain-containing 1) is a member of the nucleotide-binding oligomerization domain-like receptor family of proteins that are key organization proteins in the inflammasome. It is reported that NALP1 plays a central role in cell apoptosis, pyroptosis, inflammatory reactions and autoimmune diseases. DAC (5-aza-2-deoxycytidine) is an antitumor drug useful to lung cancer, myelodysplastic disorders, myelodysplasia and acute myeloid leukemia. In this study, we examined the expression of NALP1 in human normal and cancerous colon tissues using tissue microarray, western blot and quantitative real-time PCR and we measured the expression of NALP1 in three kinds of colon cancer cell lines and animal models before and after treatment with DAC. Furthermore, we examined the treatment effects of DAC on colon cancer in our animal model. Our data indicate that NALP1 is expressed low in human colorectal tumoral tissues relative to paratumoral tissues and was associated with the survival and tumor metastasis of patients. The expression of NALP1 increased after treatment with DAC both *in vitro* and *in vivo*. Furthermore, DAC suppressed the growth of colon cancer and increased lifespan in mouse model. Therefore, we conclude that NALP1 is expressed low in colon cancer and associated with the survival and tumor metastasis of patients, and treatment with DAC can restore NALP1 levels to suppress the growth of colon cancer.

Colorectal cancer is one of the most common forms of fatal cancer in the world,^1^ yet the molecular mechanisms underlying its growth are poorly understood.^2^ Intestinal epithelial cells play an important role in the innate defense of the intestine, and impairment of epithelial functions can result in inflammatory bowel diseases (IBD)^3, 4, 5, 6^ such as ulcerative colitis. Epidemiological studies in patients with IBD have clearly identified chronic active inflammation as a major risk factor for colon adenocarcinoma.^7, 8, 9^ Although colitis-associated colorectal cancers (CACs) comprise ^<^5% of all colorectal cancers,^10^ the cumulative incidence of CAC in patients with ulcerative colitis 25–30 years after diagnosis ranges from 8 to 43%, accounting for one-sixth of all deaths in this group.^11^ Treatment with nonsteroidal anti-inflammatory therapy reduces the risk of cancer in ulcerative colitis patients by 40–50% and reduces the risk of developing polyps in patients with familial adenomatous.^12, 13^ The data suggest that anti-inflammatory therapy with nonsteroidal anti-inflammatory drugs (NSAIDs) reduces the risk of CAC.^14^

The NALP1 (nucleotide-binding oligomerization domain-like receptor family pyrin domain-containing 1) protein, which contains a pyrin domain at its NH3 terminus and a CARD (caspase recruitment domain-containing protein) domain at its COOH terminus, has attracted recent interest in the field of CAC. NALP1 (also called CARD7 or NAC) is the first NALP-family protein to be discovered by its sequence homology to APAF-1 (apoptotic protease-activating factor-1) and has been implicated in cell responses to apoptotic and inflammatory stimuli. NALP1 is a multidomain scaffold protein that contains an N-terminal pyrin domain (PYD) followed by a centrally located NACHT domain, five tandem LRR domains, a FIIND (domain with function to find) domain and a C-terminal CARD domain.^15^ NALP1 protein is believed to interact with APAF-1 to subsequently enhance apoptosis and/or activate the proinflammatory caspases in conjunction with ASC (apoptosis-associated speck-like protein containing a CARD). ASC is an essential component of the inflammasome and connects NALP1 to caspase-1. The PYD of ASC interacts with the PYD of NALP1, and the CARD of ASC recruits the CARD of procaspase-1. Furthermore, NALP1 has been implicated in cell pyroptosis, a newly discovered form of programmed cell death.^16^

The 5-aza-2-deoxycytidine (DAC) is a DNA methylation inhibitor that has found use as an antitumor drug in mammary cancer,^17^ non-small-cell lung cancer,^18^ rhabdomyosarcoma and medulloblastoma.^19^ At low doses, DAC can exert durable antitumor effects without cytotoxicity both *in vitro* and *in vivo*.^18, 20^

Recent articles have focused on the role of NALP1 in pyroptosis and inflammation, but the expression of NALP1 in colon cancer remains unknown. Furthermore, studies examining the effects of DAC on colon cancer are rare. Considering the specific multidomain architecture of NALP1, the abnormal expression of NALP1 in some diseases and its signal-mediated role in apoptosis, we choose human colorectal carcinoma tissue as a representative example of clinical solid tumors and examined the expression of NALP1. We designed our experiment to explore the correlation between NALP1 and human colorectal carcinoma. We studied the expression of NALP1 in human normal colon tissues and colon cancer tissues and measured the expression level of NALP1 in three kinds of colon cancer cell lines in the presence or absence of DAC treatment to investigate the mechanisms underlying reduced expression of NALP1 in colon cancer and the effects of DAC treatment.

## Results

### Reduced expression of NALP1 in human colon cancer tissues

**Figure 1:**
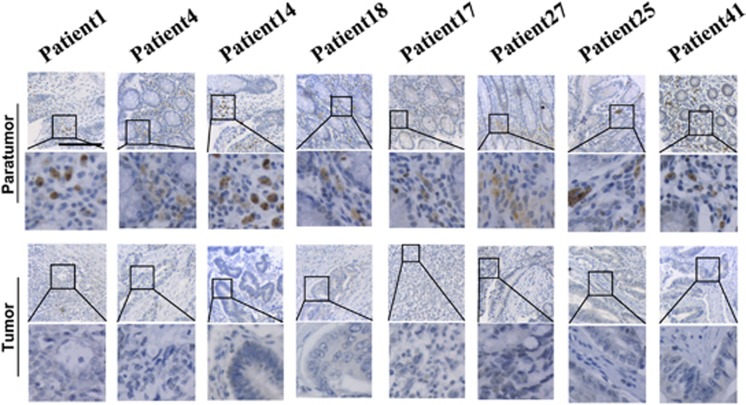
Tissue chip analysis of NALP1 expression in paratumoral tissue and tumor tissues. The cytoplasm of both glandular epithelial cells and interstitial cells stained positive for NALP1. Epithelial cells in normal colon tissues expressed more NALP1 than cancer tissues. Scale bars=50 *μ*m. According to TNM staging, patients 1 and 4 are stage I, patients 14 and 18 are stage II, patients 17 and 27 are stage III and patients 25 and 41 are stage IV. We selected two typical representative examples for each stage

We measured the expression of NALP1 in 50 colon cancer and normal tissue patient samples using a tissue microarray. The clinicopathological features of the patients are described in Table 1. The sample information is described in Supplementary Table 1. NALP1-positive cells exhibited primarily cytoplasmic staining of both glandular epithelial cells and interstitial cells between the glands. In the 34 normal tissue samples that exhibited positive staining, the para-carcinoma colonic tissue exhibited stronger NALP1 cytoplasm staining compared with the cancer tissue in 30 cases. Figure 1 depicts a representative staining example. The frequency of NALP1 expression is described in Table 2. Overall, NALP1 was expressed at lower levels in colon cancer tissues than that in normal tissues.

### NALP1 mRNA is expressed at reduced levels in human colorectal cancer tissue

**Figure 2:**
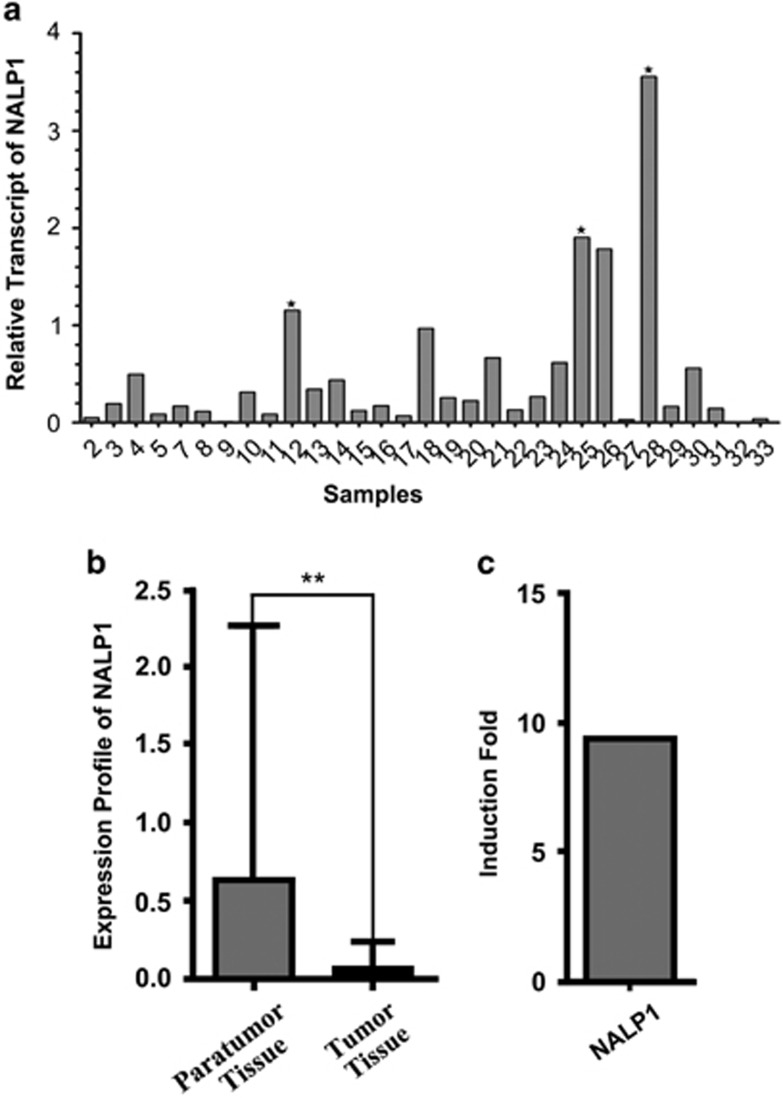
NALP1 mRNA expression in 31 human colorectal cancer tissues. (**a**) The human colorectal cancer tissue qRT-PCR array was used to measure transcript levels of NALP1 mRNA. For the human tissue array, tissues were selected from 33 individuals with cancer of different stages, except patients 1 and 6. The sample information is described in Table 1. The y axis represents the relative transcript value of NALP1 mRNA. Data were obtained using the comparative *C*_*T*_ method, and the data were normalized to GAPDH levels and the matched-pairs normal colorectal tissue. The bar with the sign (^★^) indicates a relative transcript value >1. (**b**) The transcript levels of NALP1 in human colorectal normal and cancer tissue, respectively. The qRT-PCR array was used to measure the transcript levels of NALP1 mRNA. The y axis represents the mean±S.D. for human colorectal normal and cancer tissues as individual data points using 2^-ΔCt^ [2^-(Ct NALP1-CtGAPDH)^]. The S.D. was calculated from these data. The mean±S.D. 2^-ΔCt^ for the normal and cancerous tissues was 0.616±1.642 and 0.066±0.152, respectively, and the difference was highly statistically significant as determined by Wilcoxon signed-rank nonparametric test; the *P-*values are represented by asterisks (**P<0.01). (**c**) The fold induction in NALP1 mRNA expression in the human colorectal normal group compared with the human colorectal cancer group. The fold induction is 0.616/0.066- or 9.34-fold. Details are provided in the ‘Materials and Methods' section. GAPDH, glyceraldehyde 3-phosphate dehydrogenase; qRT-PCR, quantitative real-time PCR

We collected an additional 33 patients' para-carcinoma tissue and cancer tissue samples to examine the differences in NALP1 expression at the transcript level. We measured the transcript levels of NALP1 in 31 matched-tissue pairs of colorectal cancer and para-carcinoma tissue using quantitative reverse transcription-PCR (RT-PCR). The sample information is described in Supplementary Table 2. In human colorectal cancer tissues, NALP1 mRNA was expressed at lower transcript levels compared with para-carcinoma tissue. The relative transcript profiles of each cancer tissue, presented in Figure 2a, revealed that the transcript levels of NALP1 mRNA were lower in 27/31 colorectal cancer tissues compared with the transcript level of NALP1 mRNA in para-carcinoma tissues. In Figure 2b, the NALP1 mRNA levels are presented as the mean±S.D. for human colorectal normal and cancer tissues as individual data points using the 2^-ΔCt^ [2^-(Ct NALP1-Ct GAPDH)^]. The NALP1 mRNA expression was 0.616±1.642 in para-carcinoma tissue samples and 0.066±0.152 in colorectal tissue samples, representing a highly statistically significant difference as determined by the Wilcoxon signed-rank nonparametric test. The *P-*values in the figure are represented by asterisks (*P*<0.01). As indicated in Figure 2c, the transcript level of NALP1 mRNA was decreased 9.34-fold in colorectal samples relative to para-carcinoma tissue.

### NALP1 protein levels are reduced in human colorectal cancer tissues relative to para-carcinoma tissue

**Figure 3:**
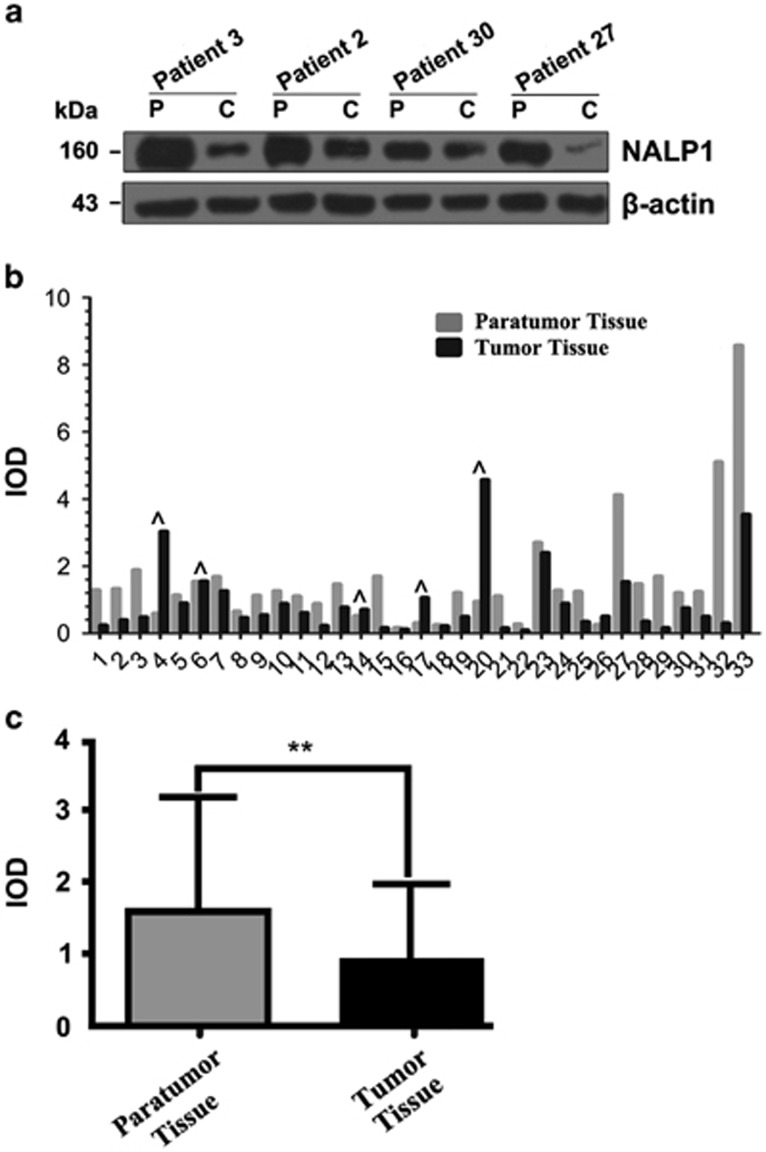
Western blot analysis of NALP1 protein expression in 33 human colorectal normal and cancer tissues. (**a**) The total soluble protein lysates (25 *μ*g) were separated by SDS-PAGE and analyzed for protein expression. The sample information is described in Table 3. For example, ‘N' and ‘C' under the heading ‘Patient 3' are represented as CL3N and CL3T in Supplementary Table 2, respectively. *β*-Actin protein expression served as a loading control. (**b**) The human colorectal cancer tissue western blot analysis was used to determine expression levels of NALP1 protein. For the human tissue array, tissues were selected from 33 matched pairs of normal and cancer tissues. The y axis represents the IOD value of NALP1 protein. Data were obtained using the Gel-Pro32 analyzer software with the values normalized to *β*-actin levels. The bar with the single sign (^) indicates that the expression value of NALP1 protein in cancer tissue is higher than normal tissue. (**c**) The expression levels of NALP1 protein in human colorectal normal and cancer tissue, respectively. The western analysis was used to determine the expression levels of NALP1 protein. The y axis represents the integrated optical density (IOD) value of NALP1 protein expression. Data were obtained using the Gel-Pro32 analyzer software with the values normalized to *β*-actin levels. The difference in the expression level of NALP1 protein in colorectal normal *versus* cancer tissue was highly statistically significant as determined by Wilcoxon signed-rank nonparametric test; the *P-*values are represented by asterisks (**P<0.01)

To investigate the expression level of NALP1 protein in human normal and cancerous colorectal tissue samples, we measured the protein level of NALP1 in 33 matched pairs of human colorectal cancer and para-carcinoma tissue. The sample information is described in Supplementary Table 2. Figure 3a depicts a typical representative western blot demonstrating NALP1 protein expression between para-carcinoma tissue and cancer in 4 of the 33 matched pairs examined in this study. The expression profiles of NALP1 protein reveal a band at ∼160 kDa, which is the reported molecular weight for NALP1 protein. The western blot results from all 33 matched-pairs patients quantified using Gel-Pro32 analyzer software are presented in the bar graph and described in Table 4. In 27 of 33 matched-pairs cases (81.8%), NALP1 protein levels were reduced in human colorectal cancer tissue relative to normal tissue. Thus, NALP1 protein expression was significantly reduced in human colorectal cancer, and NALP1 protein levels in para-carcinoma tissue were significantly elevated relative to human colorectal cancer tissue. As shown in Figure 3c, the difference in the expression level of NALP1 protein in human colorectal tissue *versus* cancer tissue was highly statistically significant as determined by the Wilcoxon signed-rank nonparametric test; the *P* values are represented by asterisks (*P*<0.01). These results above demonstrate unambiguously that NALP1 protein is expressed at low levels in human colorectal cancer tissues compared with normal tissues.

### The expression of NALP1 in stage I/II and stage III/IV colorectal cancer

**Figure 4:**
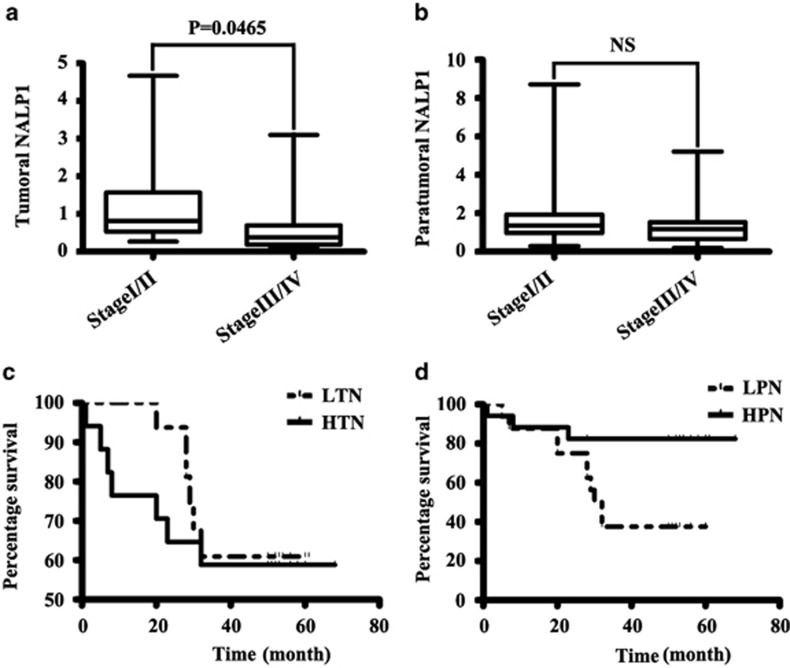
The staging and survival analyses. (**a**) T test of NALP1 expression in tumor tissue between stage I/II and stage III/IV disease. The expression of NALP1 in stage I/II was significantly higher than stage III/IV disease (*P*=0.0465). (**b**) T test of NALP1 in paratumoral tissue between stage I/II and stage III/IV disease revealed no significant difference (*P*=0.2418). (**c**) Survival analysis of 33 cases. There was no significant difference between the two groups. HTN, higher tumor NALP1; LTN, lower tumor NALP1. The *P-*value was 0.6443. (**d**) Survival analysis of 33 cases. The difference between the two groups is significant. HPN, higher paratumoral NALP1; LPN, lower paratumoral NALP1. The *P-*value was 0.0199

Next, we explored the difference in NALP1 protein expression in stage I/II and stage III/IV human colon cancer tissue. In paratumoral tissue, there was no significant difference in NALP1 expression between stage I/II and stage III/IV samples (*P*=0.2418; Figure 4a). However, the expression of NALP1 differed significantly between stage I/II and stage III/IV tumor tissues (*P*=0.0465; Figure 4b). The statistical analysis was performed using the unpaired-*t* test.

### Survival analysis

To determine whether NALP1 expression is associated with the worse clinical outcomes, we collected the survival data of the 33 patients. The relative values of NALP1 in paratumoral and tumoral tissue and the patient survival times are described in Table 4. In paratumoral tissue, the 33 patients were divided into two groups according to the relative NALP1 expression in paratumoral tissue: 16 in the group with lower NALP1 (LPN) expression and 17 in the group with higher NALP1 expression (HPN). We performed survival analysis using the log-rank (Mantel–Cox) test. The *P-*value was 0.0199, indicating a significant difference between the two groups (Figure 4c). Thus, the expression of NALP1 in paratumoral tissue was associated with the survival of patients, and the patients with higher paratumoral NALP1 exhibited longer survival times. We performed a similar analysis with respect to the tumoral NALP1 expression by dividing the samples into two groups, LTN and HTN, representing lower and higher NALP1 expression, respectively. The *P-*value was 0.6443, and there was no significant difference between the two groups (Figure 4d). The survival information and relative NALP1 values are described in Table 4.

### DAC cytotoxicity detection using the CCK-8 kit

**Figure 5:**
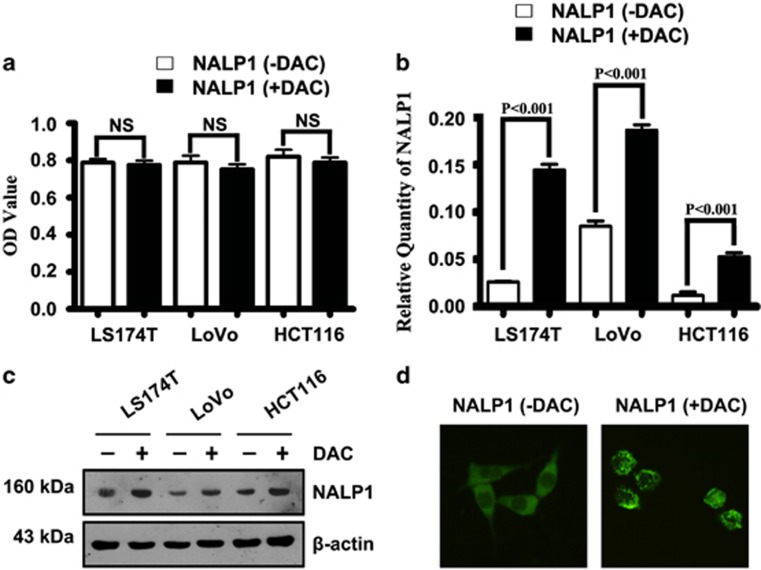
Expression of NALP1 in colon cancer cell lines before and after DAC treatment. (**a**) The cytotoxicity of DAC *in vitro*. No cytotoxicity was observed in any of the three cell lines at 1.0 *μ*M DAC. (**b**) Expression of NALP1 mRNA relative to GAPDH in three kinds of colon cancer cell lines before and after DAC treatment. (**c**) Western blot analysis of NALP1 protein before and after DAC treatment. (**d**) Representative immunofluorescence staining of NALP1 in LS174T cells (untreated with DAC, left panel; treated with DAC, right panel)

To detect the cytotoxicity of DAC, we performed a CCK-8 experiment. As shown in Figure 5a, there was no significant difference between the experimental group and negative control. We conclude that 1.0 *μ*M DAC produced no cytotoxicity in any of the three cell lines.

### Treatment with DAC increased NALP1 expression *in vitro*

We treated the colon cancer cell lines with DAC and observed the expression of NALP1 in three colon cancer cell lines using qRT-PCR, western blot and immunofluorescence. In the three cell lines, we detected increased expression of NALP1 by qRT-PCR (Figure 5b) and western blot analysis (Figure 5c) after treatment with 1 *μ*M DAC. Immunofluorescence analysis of NALP1(Figure 5d) revealed some cytoplasmic dot-like staining after treatment with DAC in the LS174T cell line in parallel with increased NALP1 expression.

### The treatment effects of DAC on colon cancer *in vivo*

**Figure 6:**
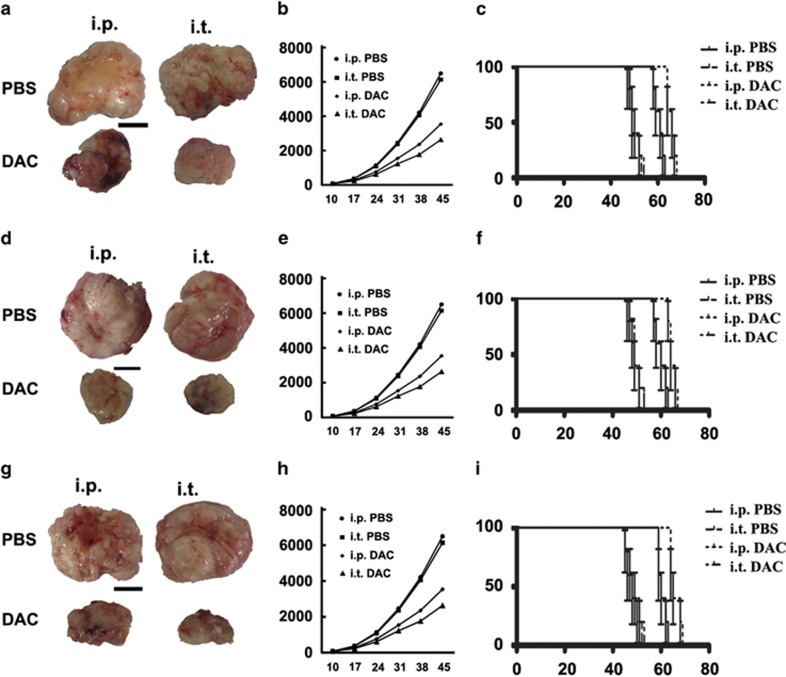
The effect of DAC treatment on colon cancer *in vivo*. (**a**, **d** and **g**) LS174T, LoVo and Hct116 tumor tissues in the murine model before and after DAC treatment. The tissues in the DAC treatment groups were much smaller than in the control groups, and the i.t. group exhibited the smallest samples. (**b**, **e** and **h**) The mean volume of LS174T, LoVo and Hct116 cancer tissues in the murine model before and after DAC treatment. DAC treatment by i.t. resulted in the smallest volumes. (**c**, **f** and **i**) The survival analysis of the LS174T, LoVo and Hct116 murine model. The drug-treated groups lived significantly longer than the drug-free groups, and the group in which DAC was i.t. injected lived longer than the group receiving i.p. injection

Treatment of established tumors with DAC resulted in a significant abrogation of tumor growth, causing almost full stasis of growth over the treatment period. Furthermore, intratumoral (i.t.) injection was more effective than intraperitoneal (i.p.) injection of DAC (Figure 6a, d and g and Figure 6b, e and h). The survival analysis revealed that the drug-treated groups survived significantly longer than the untreated groups, and the group in which DAC was i.t. injected survived longer than the group receiving i.p. injection (Figure 6c, f and i).

### A 5-aza-2′-deoxycytidine treatment increased NALP1 expression *in vivo*

**Figure 7:**
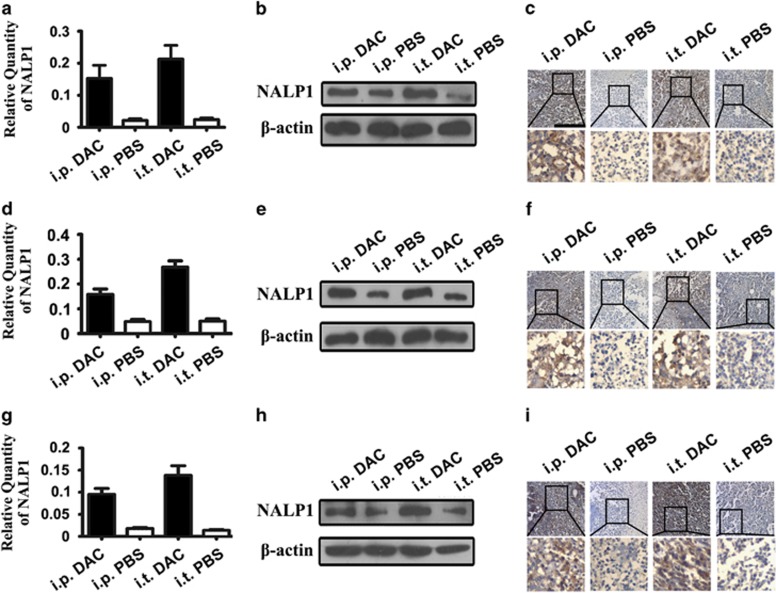
Expression of NALP1 in colon cancer cell lines before and after DAC treatment. (**a**, **d** and **g**) Expression of NALP1 mRNA relative to GAPDH in LS174T, LoVo and Hct116 tissues in the murine model before and after DAC treatment. (**b**, **e** and **h**) Western blot analysis for NALP1 in LS174T, LoVo and Hct116 tissues in the murine model before and after DAC treatment. (**c**, **f** and **i**) Immunohistochemistry analysis of NALP1 protein expression in LS174T, LoVo and Hct116 tissues in the murine model before and after DAC treatment

Nude mice inoculated with colon cancer cell were treated with DAC, and NALP1 expression was observed in three kinds of tumor tissues using RT-PCR (Figure 7a, d and g), western blot (Figure 7b, e and h) and immunohistochemistry. In the three kinds of tumor tissues, we detected increased expression of NALP1 by qRT-PCR, western blot and immunohistochemistry after treatment with DAC (Figure 7c, f and i).

### CpG islands in the *NALP1* gene

To further examine the relationship between methylation and the reduced expression of NALP1 in colon cancer as well as the results of DAC treatment, we examined the presence of CpG islands in the *NALP1* gene. DNA methylation normally occurs at cytosine residues within CpG dinucleotides. The presence of CpG islands in the *NALP1* gene was measured using Methyl Primer Express Software v1.0 (Applied System, Foster City, CA, USA). The presence of any CpG island in *NALP1* gene would suggest that methylation is the cause of the tumor-specific reduction in NALP1 expression. We confirmed that there were no CpG islands in NALP1 (data not shown), suggesting that the *NALP1* gene is not methylated. Thus, the reduced expression of NALP1 in colon cancer tissues was not the effect of methylation.

## Discussion

In this study, we have measured the expression of NALP1 in human colorectal para-carcinoma and carcinoma tissues. Furthermore, we measured the expression of NALP1 in three colon cancer cell lines before and after treatment with DAC both *in vivo* and *in vitro*. Finally, we also investigated the potential mechanism underlying reduced expression of NALP1 in colon cancer and the effects of DAC treatment. To our knowledge, this is the first report suggesting that the expression of NALP1 is reduced in colon cancer tissue relative to normal tissue and that DAC can restore the expression of NALP1 in colon cancer both *in vivo* and *in vitro* to suppress colon cancer. In addition, we are the first to report that the expression level of NALP1 in paratumoral tissue is associated with patient survival, whereas the tumoral expression of NALP1 is not.

We collected para-carcinoma and carcinoma tissue samples from 50 patients to perform immunohistochemistry analysis. We found that NALP1 is expressed at lower levels in cancer tissue. NALP1 has been reported to strongly stain glandular epithelial cells.^21^ However, in our experiment, we found that NALP1 is also expressed in interstitial cells between the glands as well as in epithelial cells.

We collected para-carcinoma and carcinoma tissue samples from an additional 33 patients and performed qRT-PCR and western blot analysis. Based on our data, NALP1 protein and mRNA levels do not correspond well with each other. Both translational and post-translational modification could be the cause of the discrepancy between NALP1 mRNA and protein levels in human colorectal tissue. In patients 12, 25 and 28 with the single sign (^★^), the transcript level of NALP1 mRNA in carcinoma tissues was higher than that in para-carcinoma tissues, but the expression level of NALP1 protein in these carcinoma tissues samples was lower than in para-carcinoma tissues; in patients 4, 6, 14, 17 and 20 with the single sign (^), the relationship between NALP1 protein expression and NALP1 mRNA expression exhibited the opposite relationship as in the patients with the single sign (^★^). This result may be because of different regulation mechanisms (such as synthesis and degradation rates) acting on both the synthesized mRNA and the synthesized protein.^22^

In paratumoral tissue, we observed no significant difference in NALP1 expression between stage I/II and stage III/IV disease, but NALP1 was associated with the survival time of patients. In tumor tissue, NALP1 levels were significantly higher in stage I/II disease compared with stage III/IV disease, but NALP1 was not associated with survival time. These data suggested that downregulation of NALP1 in tumor tissue may be a step in tumor metastasis and that the overexpression of NALP1 in para-tumoral tissue improves clinical outcomes. Therefore, we suggest that NALP1 may represent a target for colon cancer treatment and that patients with higher NALP1 expression in colon tissue may be at reduced risk of developing colon cancer.

In light of the increased expression of NALP1 in three kinds of colon cancer cell lines before and after using DAC treatment both *in vivo* and *in vitro*, we hypothesized that methylation may underlie the change in NALP1 expression. DAC is a demethylation agent and exhibits antitumor effects. After treatment with DAC, the expression of ASC increases in many kinds of tumor cells.^15^ NALP1 and ASC belong to the same protein family, and they can form an immunocomplex, activate the proinflammatory caspase and enhance the apoptotic function. Therefore, we hypothesized that DAC treatment would increase the expression of NALP1. Furthermore, we analyzed the presence of CpG islands in the *NALP1* gene. NALP1 was expressed at low levels in colon cancer cells, and its expression increased upon DAC treatment both *in vivo* and *in vitro*. However, the low level of NALP1 expression was not because of methylation, as no CpG islands were detected in the *NALP1* gene. Thus, the increase in NALP1 expression in colon cancer cell lines after drug treatment was independent of the demethylation effect of DAC. There are many potential explanations for the increase in NALP1 expression. For example, DAC may increase the expression of some associated protein that enhances NALP1 expression in colon cancer. Alternatively, DAC may activate a signaling molecule by demethylation and thereby restore NALP1 expression.

NALP1 is unique in having an N-terminal pyrin domain as well as an NBD-LRR domain and a C-terminal CARD domain. NALP1 may represent a target for cancer treatment in light of its ability to induce apoptosis and its frequent loss in various tumors.^23^ The NALP1 protein is a component of an important and large protein complex called the apoptosome,^24^ a multiprotein complex composed of Apaf-1, caspases and additional proteins.^25^ Overexpressed NALP1 can interact with caspases and Apaf-1 through a CARD–CARD homotypic interaction.^26^ NALP1 has also been reported to interact with Apaf-1 and it subsequently recruits caspases to the complex, and overexpression of NALP1 can stimulate apoptosis and enhance caspase activation induced by exogenous Apaf-1.^24^ NALP1 has also been reported to interact with apoptotic caspases such as caspase-2 and caspase-9,^24, 26^ and overexpression of NALP1 induces apoptosis in breast carcinoma cells.^26^ Liu *et al.*^27^ reported that expression of NALP1 stimulates apoptosis through activation of caspase-3. These studies suggest that overexpression of NALP1 plays an important function in apoptosis and suggest a complex interplay between NALP1 and molecules found in large complexes.

Although it has long been a focus of study, the minimal pathogenesis of colorectal carcinoma remains poorly understood. Many molecules participate in this complex procedure. Although progress has been made, there remains a great deal to be discovered. We have demonstrated here that NALP1 is expressed at reduced levels in human colorectal carcinoma tissues and that DAC can increase the expression of NALP1 both *in vivo* and *in vitro*. Furthermore, we have demonstrated that DAC can be used to treat colon cancer in an animal model. Based on our research, the striking reduction of NALP1 in human colorectal carcinoma may contribute to human colorectal carcinoma pathogenesis. We also suggest that NALP1 may represent a target for future colorectal carcinoma therapy.

## Materials and Methods

### Cell culture

Three human colon cancer cell lines were used for research: LS174T, LoVo and Hct-116. Cells were grown in 1640 culture media supplemented with 10% heat-inactivated fetal bovine serum and 1% penicillin and streptomycin. Cell lines were maintained in a humidified incubator at 37°C with an atmosphere of 5% CO_2_.

### Nude mice

Sixty nude mice were purchased from Dalian Medical University (Dalian, China). All mice were maintained in specific pathogen-free conditions. All animal experiments were performed in accordance with the national and institutional guidelines for animal care and were approved by the animal use and care committee.

### Primary colon samples

Colon tissues and tumors were obtained from surgical resection at the Second Affiliated Hospital of Dalian Medical University. All patients were preoperatively diagnosed with colon carcinoma and did not receive chemotherapy or radiation before operation. There were no ethical issues raised in this experiment. The study was approved by the Research Ethics Committee of Dalian Medical University, and informed consent was obtained from all participants in agreement with the institutional guidelines.

### Tissue microarray

A tissue microarray block was constructed with a custom-built instrument (Beecher Instruments, Silver Spring, MD, USA). The sample diameter was 600 *μ*m, and the spacing between adjacent specimens was 100 *μ*m. The tissue microarray included 50 colon cancer and 50 adjacent normal tissue samples (see Table 1).

### Immunohistochemistry

Immunohistochemistry for the NALP1 antigen was performed on paraffin sections including 5-mm microarray sections of each colon cancer tissue as described above and cancer tissues from a colon cancer model. Slides were stained with anti-NALP1 (rabbit polyclonal anti-human, 1 : 100, Santa Cruz Biotechnology, Santa Cruz, CA, USA, cat. no. 58550). Briefly, following deparaffinization, antigen retrieval for NALP1 was performed in 10 mM citrate buffer (pH 6.0) using a microwave/pressure cooker for 20 min at medium. Following H_2_O_2_ and serum blocking, slides were incubated with the primary antibody at 4°C overnight. The samples were incubated with an HRP-conjugated anti-rabbit secondary antibody (1 : 1000, Santa Cruz Biotechnology) for 45 min. The slides were then incubated with diaminobenzidine (DAB) and counterstained with hematoxylin. Microscopic examination was performed using an Olympus multifunction microscope (Olympus BX51, Tokyo, Japan). Negative controls were processed in an identical manner as positive control samples but were incubated with rabbit IgG rather than primary antibodies.

### Tissue microarray assessment

Two pathology experts evaluated the immunohistochemical staining blinded to the clinicopathological features or clinical outcome. There was a high level of correlation between the two scorers, and in the few discrepant cases, a consensus was reached after joint review. The samples were scored with respect to the percentage of cells positively stained for cytoplasmic NALP1: no intense pattern, negative (−):<10% immunoreactivity, weak (±); 10–50% immunoreactivity, moderate (+); and >50% immunoreactivity, strong (++). Scores were entered into a Microsoft Excel spreadsheet. The statistical analysis of expression of NALP1 in normal colon mucosa and cancer tissues was performed by the *χ*^2^ test (Table 2).

### Quantitative RT-PCR

Quantitative RT-PCR was performed to characterize the expression profile of human target genes using quantitative RT-PCR arrays according to the manufacturer's protocol. Quantitative RT-PCR was performed in 96-well optical plates using TP800 (TaKaRa, Shiga, Japan) with primers specific for NALP1 or glyceraldehyde 3-phosphate dehydrogenase (GAPDH) and SYBRPremix Ex Taq (TaKaRa). In brief, we extracted total mRNA from each cell line or colon cancer tissue before and after DAC treatment. We diluted the each RNA sample using RNase-free water to 2 *μ*g/*μ*l. First-strand cDNA was synthesized using PrimeScript II 1st Strand cDNA Synthesis Kit (TaKaRa) and subsequently diluted with nuclease-free water to 20 *μ*g/*μ*l. All procedures were conducted according to the manufacturer's instructions. Next, we performed quantitative PCR using a Thermal Cycler Dice Real Time System (TaKaRa Bio, Otsu, Japan). The sequences of the primers were as follows: forward primer 5′-AAGACCAGCTGTTCTCGGAGTT-3′ and reverse primer 5′-AGGCATGAGATCTCCTGGTTTC-3′ for NALP1; and forward primer 5′-GCCAAAAGGGTCATCATCTC-3′ and reverse primer 5′-GGCCATCCACAGTCTTCT-3′ for GAPDH, respectively. The total reaction volume was 25 *μ*l, including 12.5 *μ*l 2 × SYBRPremix Ex TaqTM PCR master mix, 0.5 *μ*l of 5 mM forward primer, 0.5 *μ*l of 5 mM reverse primer, 2 *μ*l (20 *μ*g/*μ*l) cDNA template and 9.5 *μ*l H_2_O. Negative controls without template were also included. GAPDH was used as an internal control. The PCR program started with 95°C for 30 s followed by 40 cycles of 95°C for 5 s and 60°C for 30 s. The final PCR step was performed to acquire the dissociation curve, validating the specificity of the PCR products. CT values were transformed to gene copy number of the template cDNA using the comparative CT method (also known as the 2-ΔΔCt method). The ΔCt is the abundance of cDNAs for transcripts of each gene normalized to GAPDH at each time point. The ΔΔCt is obtained by subtracting a calibrator value (matched pairs of human colorectal normal tissue) for each gene transcript being assayed. Melting curve analysis was used for product validation. All patients were preoperatively diagnosed with colon carcinoma and did not receive chemotherapy or radiation before the operation (see Table 3 and Supplementary Table 2).

### Western blotting

All proteins were extracted using a total protein extraction kit (KeyGENE, Nanjing, China). Tissues were digested in protein lysis buffer supplemented with protease inhibitors, phenylmethanesulfonyl fluoride and phosphatase inhibitors (KeyGENE) in a homogenizer and incubated for 30 min. The samples were kept on ice at all times and clarified by centrifugation at 5000 r.p.m. for 5 min at 4°C. Cellular proteins were extracted using the same process without homogenization. The whole-tissue lysates were boiled for 5 min in the presence of loading buffer. Equal amounts of denatured protein (25 *μ*g) were resolved by electrophoresis on a 10% sodium dodecyl sulfate (SDS)-polyacrylamide gel (PAGE) under reducing conditions and electrophoretically transferred to polyvinylidene difluoride (PVDF) membranes (Millipore, Billerica, MA, USA). The membranes were then incubated with blocking buffer (5% w/v nonfat dry milk in Tris-buffered saline containing 0.1% v/v Tween-20 (TBST)) for 2 h at 37°C and incubated overnight at 4°C with mouse anti-human NALP1 polyclonal antibodies (1 : 200) in blocking buffer and mouse anti-*β*-actin polyclonal antibodies (1 : 500) in phosphate-buffered saline (PBS) that was used as an internal control for crossreactivity with the corresponding human protein. The membranes were washed six times with TBST for 10 min per wash at room temperature, followed by incubation with the HRP (horseradish peroxidase)–conjugated anti-mouse IgG antibody (1 : 2000) in PBS (ZSGB-BIO, Peking, China) for 90 min. The membranes were washed six times with TBST, 10 min per wash, at room temperature. Finally, the membranes were exposed to X-ray films to detect the samples using an enhanced chemiluminescence development reagent (ECL system, Pierce, Thermo Fisher Scientific, Rockford, IL, USA) according to the manufacturer's instructions, and the photographs of the immunoreactive bands were obtained using a HP scanner (Andover, MA, USA). Signal intensities were quantified using Gel-Pro32 analyzer software. The relative expression ratio of NALP1 was calculated as follows: relative expression ratio of NALP1 integrated optical density (IOD)/*β*-actin IOD. All patients were preoperatively diagnosed with colon carcinoma and were not treated with chemotherapy or radiation before operation (see Table 3 and Supplementary Table 2).

### DAC cytotoxicity detection

A CCK-8 kit was used to detect the cytotoxicity of 1 *μ*M DAC *in vitro*. Three groups were established, blank, negative control and experimental group, each with four wells. A 96-well plate was seeded with 100 *μ*l cell suspensions containing 1 × 10^4^ cells/well for the negative control and experimental group, and media containing no cells were used for the blank group. The cells were cultured for 24 h (37°C, 5% CO_2_). DAC solution (10 *μ*l) was added to the experimental groups to a final concentration of 1 *μ*M, and 10 *μ*l PBS was added to the other two groups. The 96-well plate was maintained in a humidified incubator at 37°C with an atmosphere of 5% CO_2_ for 48 h. Subsequently, 10 *μ*l CCK-8 solution was added to every well in each group, and the cells were cultured for 4 h. The optical density (OD) value was measured with a microplate reader at 450-nm wavelength.

### DAC treatment *in vitro*

For drug treatments, 1 × 10^5^ cells were plated in growth medium. At 24 h after plating, cultured cells were treated with a concentration of 1 *μ*M DAC (Sigma, San Francisco, CA, USA)^28^ for 72 h. The media were removed and replaced with fresh media every 24 h for a period of 72 h. After DAC treatment, cells were harvested and DNA was collected via the aforementioned extraction method.

### Immunofluorescence

Approximately 1 × 10^5^ cells were plated in growth medium. At 24 h after plating, cells were treated with 1.0 *μ*M DAC (Sigma) for 48 h with media changes every 12 h. Treated and mock-treated cells were fixed with ice-cold methanol for 15 min at 20°C or 2% paraformaldehyde in 1 × PBS, pH 7.0. Cells were washed with 1 × PBS (pH 7.0), incubated with diluted primary antibody (in 1 × PBS with 0.1% Tween-20) for 1 h at room temperature, washed three times with PBST and subsequently incubated with the appropriate fluorophore-conjugated secondary antibody in PBST. A monoclonal antibody against human NALP1 was used as the primary antibody (R&D, Minneapolis, MN, USA). Anti-mouse IgG antibody (Zhong Shan Goldenbridge Biotechnology Co., Ltd, Beijing, China) was used as the secondary antibody. Finally, the cells were examined under a fluorescence microscope (Olympus).

### The detection of CpG islands

We detected CpG islands in the *NALP1* gene by Methyl Primer Express Software v1.0 (Applied System). Briefly, we obtained the gene sequence of NALP1 and then imported the sequence into the software. Then, we clicked ‘Find CpG islands' in the drop-down menu.

### DAC treatment *in vivo*

In our experiments, we used 20 mice for each of 3 cell lines for a total of 60 mice. Approximately 1 × 10^6^ Lovo, LS174T or HCT116 cells were injected subcutaneously into the right flank of 20 nude mice. When the tumors became visible (80 mm^3^), the mice were randomly divided into four groups with five mice per group. DAC treatment was initiated by either i.p. or i.t. injection. Five mice were used per treatment group. DAC was injected i.p. or i.t. three times on days 2, 4 and 7 at a dose of 0.1 mg/kg/day without cytotoxicity.^18^ DAC was dissolved in PBS. The control group received only PBS. Tumor volumes were measured three times a week before DAC treatment and were calculated as follows: width^2^ × length × 0.52.

### Survival analysis

We recorded the survival time of 33 cases whose information is described in Table 4. The date of natural death of mice was also recorded, and we calculated the survival of mice from the day of inoculation with cancer cells. The statistical software GraphPad Prism5 (GraphPad Software, Inc., San Diego, CA, USA) was used to perform survival analysis.

### Statistical analysis

To clarify the ability to distinguish controls from patient cases, NALP1 levels were evaluated using the *χ*^2^ test. Pairwise comparisons between two groups were also performed. Statistical significance was determined using the Kruskal–Wallis nonparametric test for more than two groups. Survival analysis was performed using the log-rank (Mantel–Cox) test. In all tests, two-sided *P-*values of <0.05 were considered significant. All statistical analyses were performed using GraphPad Prism5.

## Figures and Tables

**Table 1:** Clinicopathological characteristics of 50 colonic cancer patients

**Group**	***N***
*Gender*	
Male	26
Female	24
	
*Age (year)*	
≤50	16
>50	34
	
*Lymph node metastasis*	
No	38
Yes	12
	
*Distant metastasis*	
No	48
Yes	2
	
*Stage*	
I	8
II	30
III	10
IV	2
	
*Location*	
Colon	50
Rectum	0

Clinicopathological characteristics of 50 colonic cancer patients whose paratumoral and tumor tissues were used for the tissue chip

**Table 2:** Expression of NALP-1 in normal colon mucosa and cancer tissues

**Tissue sample**	**Expression of NALP-1**					***P*** (***χ***^**2**^ **test)**
	***N***	**−**	**±**	**+**	**++**	
Normal mucosa	50	14	16	16	4	<0.001*
Colon cancer	50	33	14	3	0	

*P*-value is based on *χ*^2^ test

NALP1 expression of clinical tissue samples was scored as follows: no intense pattern, negative (−); <10% immunoreactivity, weak (±); 10–50% immunoreactivity, moderate (+); and >50% immunoreactivity, strong (++)

*Significant difference

**Table 3:** Summary of clinicopathological features of tissues used in this study

**Group**	**Number**
*Gender*	
Male	12
Female	21
	
*Age (year)*	
≤50	6
>50	27
	
*Lymph node metastasis*	
No	19
Yes	14
	
*Distant metastasis*	
No	25
Yes	8
	
*Stage*	
I	3
II	12
III	13
IV	5
	
*Location*	
Colon	18
Rectum	15

Clinicopathological characteristics of 33 colonic cancer patients whose paratumoral and tumor tissues were used for qRT-PCR and western blot

**Table 4:** The survival information and relative NALP1 value

**Patient no.**	**NALP1 of paratumor**	**NALP1 of tumor**	**Survival date**	**Death/live**
1	1.35	0.27	61	0
2	1.35	0.43	60	0
3	1.93	0.52	60	0
4	0.62	3.1	20	1
5	1.17	0.94	32	1
6	1.59	1.6	8	1
7	1.73	1.3	68	0
8	0.68	0.51	20	1
9	1.17	0.57	7	1
10	1.3	0.9	1	1
11	1.14	0.63	5	1
12	0.92	0.25	53	0
13	1.51	0.81	23	1
14	0.55	0.75	50	0
15	1.75	0.19	28	0
16	0.19	0.14	32	1
17	0.33	1.1	51	0
18	0.27	0.24	28	1
19	1.25	0.52	30	1
20	0.97	4.67	60	0
21	1.14	0.19	29	1
22	0.3	0.11	28	1
23	2.78	2.46	60	0
24	1.32	0.92	53	0
25	1.29	0.38	56	0
26	0.29	0.54	56	0
27	4.21	1.57	56	0
28	1.51	0.38	50	0
29	1.75	0.19	52	0
30	1.25	0.79	52	0
31	1.29	0.53	52	0
32	5.21	0.33	54	0
33	8.71	3.62	58	0

The survival information and relative NALP1 expression of the 33 clinical cases. We recorded the survival time and survival state of every case as well as the relative NALP1 expression in paratumoral tissue and tumor tissue

## Abbreviations

DAC: 5-aza-2-deoxycytidine
NALP1: nucleotide-binding oligomerization domain-like receptor family, pyrin domain-containing 1
IBD: inflammatory bowel diseases
CAC: colorectal cancer
NSAID: nonsteroidal anti-inflammatory drug
CARD: caspase recruitment domain-containing protein
APAF-1: apoptotic protease-activating factor-1
PYD: pyrin domain
LRR: leucine-rich repeat
FIIND: domain with function to find
ASC: apoptosis-associated speck-like protein containing a CARD
RT-PCR: reverse transcription-PCR
GAPDH: glyceraldehyde-3-phosphate dehydrogenase
SDS: sodium dodecyl sulfate
PAGE: polyacrylamide gel
PVDF: polyvinylidene difluoride
PBS: phosphate-buffered saline
TBST: Tris-buffered saline and Tween-20
HRP: horseradish peroxidase
IOD: integrated optical density
OD: optical density
i.p.: intraperitoneal
i.t.: intratumoral
LPN: lower paratumoral NALP1
HPN: higher paratumoral NALP1
LTN: lower tumor NALP1
HTN: higher tumor NALP1
