# Supplementary material for: DAC can restore expression of NALP1 to suppress tumor growth in colon cancer
Source: Cell Death Dis. 2015 Jan 22;6(1):e1602–. doi: 10.1038/cddis.2014.532 (PMC4669739; doi:10.1038/cddis.2014.532)
Supplement: Supplementary Table 1 [file cddis2014532x1.doc]

Supplement-table1 Clinicopathological characteristics of 50 colon cancer patients

| **Tumor** | **Sample** | **TNM staging** | **Appearance** | **Age/Gender** | **Pathologic Diagnosis** |
| --- | --- | --- | --- | --- | --- |
| **1** | CL1N |  | Adjacent Normal | 67/F. | Normal |
|  | CL1P | T2N0M0 | Primary Tumor |  | Adenocarcinoma |
| **2** | CL2N |  | Adjacent Normal | 70/M. | Normal |
|  | CL2P | T4N1M0 | Primary Tumor |  | Adenocarcinoma |
| **3** | CL3N |  | Adjacent Normal | 40/M. | Normal |
|  | CL3P | T3N0M0 | Primary Tumor |  | Adenocarcinoma |
| **4** | CL4N |  | Adjacent Normal | 45/F. | Normal |
|  | CL4P | T2N0M0 | Primary Tumor |  | Adenocarcinoma |
| **5** | CL5N |  | Adjacent Normal | 73/M. | Normal |
|  | CL5P | T4N0M0 | Primary Tumor |  | Adenocarcinoma |
| **6** | CL6N |  | Adjacent Normal | 61/M. | Normal |
|  | CL6P | T4N0M0 | Primary Tumor |  | Adenocarcinoma |
| **7** | CL7N |  | Adjacent Normal | 55/F. | Normal |
|  | CL7P | T3N1M0 | Primary Tumor |  | Adenocarcinoma |
| **8** | CL8N |  | Adjacent Normal | 73/M. | Normal |
|  | CL8P | T4N0M0 | Primary Tumor |  | Adenocarcinoma |
| **9** | CL9N |  | Adjacent Normal | 73/M. | Normal |
|  | CL9P | T3N0M0 | Primary Tumor |  | Adenocarcinoma |
| **10** | CL10N |  | Adjacent Normal | 60/F. | Normal |
|  | CL10P | T2N0M0 | Primary Tumor |  | Adenocarcinoma |
| **11** | CL11N |  | Adjacent Normal | 32/F. | Normal |
|  | CL11P | T3N0M0 | Primary Tumor |  | Adenocarcinoma |
| **12** | CL12N |  | Adjacent Normal | 62/F. | Normal |
|  | CL12P | T3N0M0 | Primary Tumor |  | Adenocarcinoma |
| **13** | CL13N |  | Adjacent Normal | 47/M. | Normal |
|  | CL13P | T4N1M0 | Primary Tumor |  | Adenocarcinoma |
| **14** | CL14N |  | Adjacent Normal | 62/F. | Normal |
|  | CL14P | T4N0M0 | Primary Tumor |  | Adenocarcinoma |
| **15** | CL15N |  | Adjacent Normal | 80/M. | Normal |
|  | CL15P | T4N1M0 | Primary Tumor |  | Adenocarcinoma |
| **16** | CL16N |  | Adjacent Normal | 43/M. | Normal |
|  | CL16P | T3N0M0 | Primary Tumor |  | Adenocarcinoma |
| **17** | CL17N |  | Adjacent Normal | 61/M. | Normal |
|  | CL17P | T3N1M0 | Primary Tumor |  | Adenocarcinoma |
| **18** | CL18N |  | Adjacent Normal | 82/F. | Normal |
|  | CL18P | T3N0M0 | Primary Tumor |  | Adenocarcinoma |
| **19** | CL19N |  | Adjacent Normal | 58/F. | Normal |
|  | CL19P | T4N0M0 | Primary Tumor |  | Adenocarcinoma |
| **20** | CL20N |  | Adjacent Normal | 74/M. | Normal |
|  | CL20P | T3N1M0 | Primary Tumor |  | Adenocarcinoma |
| **21** | CL21N |  | Adjacent Normal | 62/F. | Normal |
|  | CL21P | T4N1M0 | Primary Tumor |  | Adenocarcinoma |
| **22** | CL22N |  | Adjacent Normal | 62/F. | Normal |
|  | CL22P | T1N0M0 | Primary Tumor |  | Adenocarcinoma |
| **23** | CL23N |  | Adjacent Normal | 54/F. | Normal |
|  | CL23P | T4N1M0 | Primary Tumor |  | Adenocarcinoma |
| **24** | CL24N |  | Adjacent Normal | 41/M. | Normal |
|  | CL24P | T4N0M0 | Primary Tumor |  | Adenocarcinoma |
| **25** | CL25N |  | Adjacent Normal | 70/F. | Normal |
|  | CL25P | T4N1M1 | Metastatic Tumor |  | Adenocarcinoma |
| **26** | CL26N |  | Adjacent Normal | 64/M. | Normal |
|  | CL26P | T4N0M0 | Primary Tumor |  | Adenocarcinoma |
| **27** | CL27N |  | Adjacent Normal | 52/F. | Normal |
|  | CL27P | T3N1M0 | Primary Tumor |  | Adenocarcinoma |
| **28** | CL28N |  | Adjacent Normal | 74/M. | Normal |
|  | CL28P | T4N0M0 | Primary Tumor |  | Adenocarcinoma |
| **29** | CL29N |  | Adjacent Normal | 57/M. | Normal |
|  | CL29P | T4N0M0 | Primary Tumor |  | Adenocarcinoma |
| **30** | CL30N |  | Adjacent Normal | 48/M. | Normal |
|  | CL30P | T2N0M0 | Primary Tumor |  | Adenocarcinoma |
| **31** | CL31N |  | Adjacent Normal | 74/F. | Normal |
|  | CL31P | T4N0M0 | Primary Tumor |  | Adenocarcinoma |
| **32** | CL32N |  | Adjacent Normal | 34/F. | Normal |
|  | CL32P | T1N0M0 | Primary Tumor |  | Adenocarcinoma |
| **33** | CL33N |  | Adjacent Normal | 31/F. | Normal |
|  | CL33P | T2N0M0 | Primary Tumor |  | Adenocarcinoma |
| **34** | CL34N |  | Adjacent Normal | 62/F. | Normal |
|  | CL34P | T4N0M0 | Primary Tumor |  | Adenocarcinoma |
| **35** | CL35N |  | Adjacent Normal | 70/F. | Normal |
|  | CL35P | T4N0M0 | Primary Tumor |  | Adenocarcinoma |
| **36** | CL36N |  | Adjacent Normal | 60/M. | Normal |
|  | CL36P | T4N0M0 | Primary Tumor |  | Adenocarcinoma |
| **37** | CL37N |  | Adjacent Normal | 69/M. | Normal |
|  | CL37P | T3N0M0 | Primary Tumor |  | Adenocarcinoma |
| **38** | CL38N |  | Adjacent Normal | 26/F. | Normal |
|  | CL38P | T3N0M0 | Primary Tumor |  | Adenocarcinoma |
| **39** | CL39N |  | Adjacent Normal | 82/M. | Normal |
|  | CL39P | T4N0M0 | Primary Tumor |  | Adenocarcinoma |
| **40** | CL40N |  | Adjacent Normal | 54/M. | Normal |
|  | CL40P | T3N0M0 | Primary Tumor |  | Adenocarcinoma |
| **41** | CL41N |  | Adjacent Normal | 47/M | Normal |
|  | CL41P | T4N2M1 | Metastatic Tumor |  | Adenocarcinoma |
| **42** | CL42N |  | Adjacent Normal | 44/M. | Normal |
|  | CL42P | T4N0M0 | Primary Tumor |  | Adenocarcinoma |
| **43** | CL43N |  | Adjacent Normal | 34/F. | Normal |
|  | CL43P | T4N0M0 | Primary Tumor |  | Adenocarcinoma |
| **44** | CL44N |  | Adjacent Normal | 40/M. | Normal |
|  | CL44P | T3N0M0 | Primary Tumor |  | Adenocarcinoma |
| **45** | CL45N |  | Adjacent Normal | 64/M. | Normal |
|  | CL45P | T3N0M0 | Primary Tumor |  | Adenocarcinoma |
| **46** | CL46N |  | Adjacent Normal | 66/F. | Normal |
|  | CL46P | T4N0M0 | Primary Tumor |  | Adenocarcinoma |
| **47** | CL47N |  | Adjacent Normal | 55/M. | Normal |
|  | CL47P | T2N0M0 | Primary Tumor |  | Adenocarcinoma |
| **48** | CL48N |  | Adjacent Normal | 81/F. | Normal |
|  | CL48P | T3N0M0 | Primary Tumor |  | Adenocarcinoma |
| **49** | CL49N |  | Adjacent Normal | 42/F. | Normal |
|  | CL49P | T4N2M0 | Primary Tumor |  | Adenocarcinoma |
| **50** | CL50N |  | Adjacent Normal | 35/M. | Normal |
|  | CL50P | T4N0M0 | Primary Tumor |  | signet-ringcell carcinoma |
|  | |  | | | |
