# Supplementary material for: DAC can restore expression of NALP1 to suppress tumor growth in colon cancer
Source: Cell Death Dis. 2015 Jan 22;6(1):e1602–. doi: 10.1038/cddis.2014.532 (PMC4669739; doi:10.1038/cddis.2014.532)
Supplement: Supplementary Table 2 [file cddis2014532x2.doc]

**Supplement-table 2: Clinicopathological Features of Tissues Used in this Study.**

| **Tumor** | **Sample** | **Tissue** | **Pathologic TNM Staging** | **Appearance** | **Age/gender** | **Pathologic Diagnosis** |
| --- | --- | --- | --- | --- | --- | --- |
| 1 | CL1N | Colon |  | Adjacent Normal | 75/F. same patient | Normal |
|  | CL1P | Colon | T2N0M0 | Primary Tumor |  | Adenocarcinoma |
| 2 | CL2N | Colon |  | Adjacent Normal | 76/M. same patient | Normal |
|  | CL2P | Colon | T3N0M0 | Primary Tumor |  | Adenocarcinoma |
| 3 | CL3N | Colon |  | Adjacent Normal | 80/M. same patient | Normal |
|  | CL3P | Colon | T4N0M0 | Primary Tumor |  | Adenocarcinoma |
| 4 | CL4N | Colon |  | Adjacent Normal | 73/F. same patient | Normal |
|  | CL4P | Colon | T3N2M0 | Primary Tumor |  | Adenocarcinoma |
| 5 | CL5N | Colon |  | Adjacent Normal | 40/F. same patient | Normal |
|  | CL5P | Colon | T4N1M0 | Primary Tumor |  | Adenocarcinoma |
| 6 | CL6N | Colon |  | Adjacent Normal | 66/F. same patient | Normal |
|  | CL6P | Colon | T3N1M0 | Primary Tumor |  | Adenocarcinoma |
| 7 | CL7N | Colon |  | Adjacent Normal | 38/M. same patient | Normal |
|  | CL7P | Colon | T4N0M0 | Primary Tumor |  | Adenocarcinoma |
| 8 | CL8N | Colon |  | Adjacent Normal | 55/M. same patient | Normal |
|  | CL8P | Colon | T4N2M0 | Primary Tumor |  | Adenocarcinoma |
| 9 | CL9N | Colon |  | Adjacent Normal | 71/M. same patient | Normal |
|  | CL9P | Colon | T2N1M1 | Primary Tumor |  | Adenocarcinoma |
| 10 | CL10N | Colon |  | Adjacent Normal | 72/F. same patient | Normal |
|  | CL10P | Colon | T4N2M1 | Metastatic Tumor |  | Adenocarcinoma |
| 11 | CL11N | Colon |  | Adjacent Normal | 68/M. same patient | Normal |
|  | CL11P | Colon | T4N2M1 | Primary Tumor |  | Adenocarcinoma |
| 12 | CL12N | Colon |  | Adjacent Normal | 67/F. same patient | Normal |
|  | CL12P | Colon | T3N1M0 | Primary Tumor |  | Signet-ring cell carcinoma |
| 13 | CL13N | Colon |  | Adjacent Normal | 80/F. same patient | Normal |
|  | CL13P | Colon | T2N0M0 | Primary Tumor |  | Moderately differentiated adenocarcinoma |
| 14 | CL14N | Colon |  | Adjacent Normal | 72/F. same patient | Normal |
|  | CL14P | Colon | T3N0M0 | Primary Tumor |  | Poorly differentiated adenocarcinoma |
| 15 | CL15N | Colon |  | Adjacent Normal | 78/F. same patient | Normal |
|  | CL15P | Colon | T3N0M0 | Primary Tumor |  | Moderately differentiated adenocarcinoma |
| 16 | CL16N | Colon |  | Adjacent Normal | 71/F. same patient | Normal |
|  | CL16P | Colon | T4N1M0 | Primary Tumor |  | Moderately differentiated adenocarcinoma |
| 17 | CL17N | Colon |  | Adjacent Normal | 76/F. same patient | Normal |
|  | CL17P | Colon | T3N0M0 | Primary Tumor |  | Poorly-Moderately differentiated adenocarcinoma |
| 18 | CL18N | Colon |  | Adjacent Normal | 58/F. same patient | Normal |
|  | CL18P | Colon | T4N2M0 | Primary Tumor |  | Moderately differentiated adenocarcinoma |
| 19 | RE1N | Rectum |  | Adjacent Normal | 63/M. same patient | Normal |
|  | RE1P | Rectum | T4N2M1 | Primary Tumor |  | Adenocarcinoma |
| 20 | RE2N | Rectum |  | Adjacent Normal | 73/F. same patient | Normal |
|  | RE2P | Rectum | T3N0M0 | Primary Tumor |  | Adenocarcinoma |
| 21 | RE3N | Rectum |  | Adjacent Normal | 59/F. same patient | Normal |
|  | RE3P | Rectum | T4N2M0 | Primary Tumor |  | Signet-ring cell carcinoma |
| 22 | RE4N | Rectum |  | Adjacent Normal | 51/M. same patient | Normal |
|  | RE4P | Rectum | T4N1M1 | Primary Tumor |  | Adenocarcinoma |
| 23 | RE5N | Rectum |  | Adjacent Normal | 65/M. same patient | Normal |
|  | RE5P | Rectum | T4N0M0 | Primary Tumor |  | Adenocarcinoma |
| 24 | RE6N | Rectum |  | Adjacent Normal | 80/F. same patient | Normal |
|  | RE6P | Rectum | T4N0M0 | Primary Tumor |  | Moderately differentiated adenocarcinoma |
| **25** | RE7N | Rectum |  | Adjacent Normal | 66/M. same patient | Normal |
|  | RE7P | Rectum | T4N2M0 | Primary Tumor |  | Moderately differentiated adenocarcinoma |
| 26 | RE8N | Rectum |  | Adjacent Normal | 87/F. same patient | Normal |
|  | RE8P | Rectum | T4N0M0 | Primary Tumor |  | Moderately differentiated adenocarcinoma |
| 27 | RE9N | Rectum |  | Adjacent Normal | 69/F. same patient | Normal |
|  | RE9P | Rectum | T3N0M0 | Primary Tumor |  | Moderately differentiated adenocarcinoma |
| 28 | RE10N | Rectum |  | Adjacent Normal | 50/F. same patient | Normal |
|  | RE10P | Rectum | T3N1M0 | Primary Tumor |  | Moderately differentiated adenocarcinoma |
| 29 | RE11N | Rectum |  | Adjacent Normal | 65/F. same patient | Normal |
|  | RE11P | Rectum | T3N1M0 | Primary Tumor |  | Moderately differentiated adenocarcinoma |
| 30 | RE12N | Rectum |  | Adjacent Normal | 45/F. same patient | Normal |
|  | RE12P | Rectum | T3N2M0 | Primary Tumor |  | Moderately differentiated adenocarcinoma |
| 31 | RE13N | Rectum |  | Adjacent Normal | 46/M. same patient | Normal |
|  | RE13P | Rectum | T3N0M0 | Primary Tumor |  | Moderately differentiated adenocarcinoma |
| 32 | RE14N | Rectum |  | Adjacent Normal | 47/M. same patient | Normal |
|  | RE14P | Rectum | T1N2M0 | Primary Tumor |  | Moderately differentiated adenocarcinoma |
| 33 | RE15N | Rectum |  | Adjacent Normal | 62/F. same patient | Normal |
|  | RE15P | Rectum | T2N0M0 | Primary Tumor |  | Poorly differentiated adenocarcinoma |
|  |  |  |  |  |  |  |
